# Cocaine- and Levamisole-Induced Vasculitis (CLIV)

**DOI:** 10.7759/cureus.92701

**Published:** 2025-09-19

**Authors:** Zahra Vaezi, Afshin Amini

**Affiliations:** 1 Internal Medicine, St. Luke's Hospital, Chesterfield, USA

**Keywords:** anca-associated vasculitis, cocaine- and levamisole-induced vasculitis, crescentic glomerulonephritis (rpgn), diffuse alveolar hemorrhage, vasculitis

## Abstract

Cocaine- and levamisole-induced vasculitis (CLIV) is a distinct form of drug-induced vasculitis characterized by dual antineutrophil cytoplasmic antibody (ANCA) positivity, rapidly progressive glomerulonephritis (RPGN), and diffuse alveolar hemorrhage (DAH). This systematic review synthesizes data from case reports, case series, and reviews published between 2015 and 2024 to clarify clinical presentations, diagnostic approaches, and management strategies. A comprehensive search of PubMed and Google Scholar using terms such as “levamisole-induced vasculitis,” “cocaine-associated vasculitis,” “dual ANCA vasculitis,” “RPGN,” and “DAH” identified 312 records. After removing 84 duplicates, 228 records were screened, 52 were assessed for eligibility, and 18 were included in the final analysis. Most patients presented with cutaneous, renal, and pulmonary involvement. Diagnostic tools included ANCA serology (MPO and PR3), biopsy, and imaging. Treatment most often involved corticosteroids, with severe cases requiring rituximab, cyclophosphamide, or plasmapheresis. Prognosis improved with early diagnosis and cessation of exposure. Recognition of CLIV in cocaine users presenting with ANCA-positive vasculitis is essential for early intervention and improved outcomes.

## Introduction and background

Drug-induced vasculitis (DIV) is a rare but significant subtype of vasculitis, typically associated with small-vessel involvement and triggered by various pharmacologic or illicit substances [1]. Among these, levamisole-adulterated cocaine has emerged as a key contributor to DIV, leading to a condition now widely recognized as cocaine- and levamisole-induced vasculitis (CLIV) [2,3]. Levamisole, once used as an antihelminthic agent and immunomodulator, is commonly added to cocaine to increase bulk and enhance stimulant effects [2,4]. However, its immunologic side effects include the development of antineutrophil cytoplasmic antibody (ANCA)-positive vasculitis, leukocytoclastic vasculitis, and thrombotic vasculopathy [5-7]. The co-administration of cocaine and levamisole is linked to endothelial injury, neutrophil activation, and immune complex deposition-pathogenic mechanisms shared with primary ANCA-associated vasculitis (AAV) [6,8]. CLIV has been increasingly reported over the past decade, with most affected patients presenting with painful purpura, skin necrosis, or retiform purpura, commonly involving the ears, face, and extremities [9,10]. Systemic manifestations may include pulmonary hemorrhage, rapidly progressive glomerulonephritis (RPGN), and rare central nervous system (CNS) involvement [11-13]. The overlap in clinical features between CLIV and idiopathic AAV often results in diagnostic delays and mismanagement [14-16]. Despite the growing body of literature, awareness of CLIV remains suboptimal. Many patients are initially treated for primary vasculitis without adequate history-taking or toxicology screening [5,17]. A better understanding of the epidemiology, pathogenesis, and treatment of CLIV is essential to guide clinicians in early diagnosis and appropriate management [2,18]. This review synthesizes data from published case reports, series, and reviews from 2015 to 2024 to provide a comprehensive overview of CLIV, with particular attention to its clinical spectrum, diagnostic markers, treatment approaches, and outcomes.

## Review

**Figure 1:**
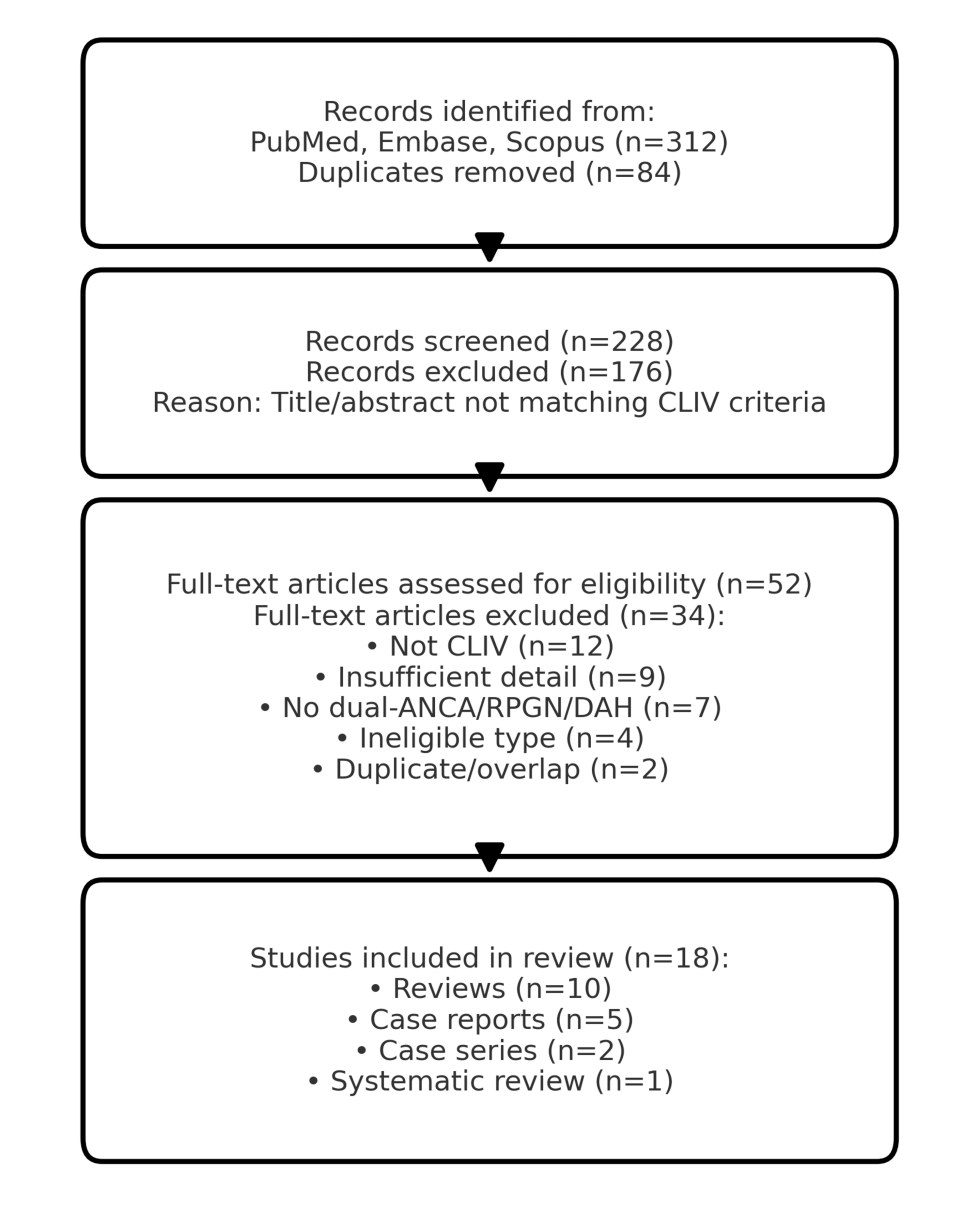
PRISMA 2020 flow diagram of study selection. CLIV: cocaine- and levamisole-induced vasculitis; ANCA: antineutrophil cytoplasmic antibody; RPGN: rapidly progressive glomerulonephritis; DAH: diffuse alveolar hemorrhage; PRISMA: Preferred Reporting Items for Systematic reviews and Meta-Analyses

We conducted a systematic search of PubMed/MEDLINE, Embase, Scopus, and CENTRAL from January 1, 2015, through July 31, 2024: PubMed (final strategy): ((levamisole[Title/Abstract] OR levamisole-adulterated[Title/Abstract]) AND cocaine[Title/Abstract]) AND (vasculitis[Title/Abstract] OR vasculopathy[Title/Abstract] OR ANCA[Title/Abstract]) with filters: Humans; English; 2015-2024. Database-specific controlled vocabulary and syntax were used for Embase (Emtree) and Scopus; full strings, run dates, and hit counts are in Supplemental material 1. We also screened the reference lists of the included reports. CENTRAL returned no eligible randomized trials. The inclusion criteria include primary reports of cocaine- and/or levamisole-associated vasculitis with dual ANCA, RPGN, and/or diffuse alveolar hemorrhage (DAH) (case reports/series/cohorts). The exclusion criteria include non-CLIV vasculitides, insufficient clinical detail, ineligible article type, and duplicates/overlaps (see the Preferred Reporting Items for Systematic reviews and Meta-Analyses (PRISMA) [19] flow diagram in Figure 1 and Supplemental materials 1, 2).

Two reviewers screened the titles/abstracts; one reviewer performed full-text extraction with senior adjudication for uncertainties. We extracted demographics, exposure confirmation (toxicology vs. self-report), organ involvement, ANCA serology (MPO, PR3), biopsy type/pathology, imaging, treatment, outcomes, follow-up, and abstinence/relapse. Risk of bias (RoB) [20] was assessed with JBI [21] checklists for case reports and case series; ROBINS-I was planned for any non-randomized comparative designs. Certainty of evidence was appraised with GRADE [22]. RoB and GRADE summaries appear in Supplemental material 3. Primary outcomes include (1) renal remission: ≥50% reduction in serum creatinine from peak or dialysis independence by 4-12 weeks; (2) pulmonary remission: cessation of DAH and oxygen independence; and (3) cutaneous response: re-epithelialization or resolution of retiform purpura/necrosis by clinical assessment. The secondary outcomes include relapse (any recurrence after initial response), need for plasmapheresis, ICU admission, and adverse events. Abstinence status was recorded when reported (toxicology-confirmed vs. patient-reported). Given the heterogeneous designs and outcomes and sparse comparative data, we did not perform quantitative pooling. We present a structured narrative synthesis of primary studies and a separate narrative of secondary reviews. Where outcomes were variably defined or missing, we report ranges and qualitatively explore sources of heterogeneity (study design, exposure confirmation). Data extraction included demographics, organ involvement, ANCA status, diagnostic methods, treatment, and outcomes. RoB was assessed qualitatively; case reports and small series had inherent selection bias, while reviews varied in search rigor. Eighteen studies met the inclusion criteria (Table 1).

**Table 1:** Literature matrix. ANCA: antineutrophil cytoplasmic antibody; CNS: central nervous system

Author(s)	Year	Study type	Organ involvement	ANCA status
Yaseen et al. [1]	2022	Review	Systemic	Dual ANCA+
Iorio et al. [2]	2024	Review	Renal, pulmonary	Dual ANCA+
Marquez et al. [3]	2017	Review	Skin, renal	Dual ANCA+
Berman et al. [4]	2016	Review	Skin	Variable
Ruffer et al. [5]	2023	Review	Systemic	Dual ANCA+
Misra et al. [6]	2021	Review	Renal	MPO/PR3+
Younger [7]	2021	Review	CNS	Not reported
Puac-Polanco et al. [8]	2024	Review	CNS	Not reported
Underner et al. [9]	2020	Review	Pulmonary	Variable
Grau [10]	2015	Review	Systemic	Dual ANCA+
Subesinghe et al. [11]	2018	Case series	Renal, pulmonary	Dual ANCA+
Roberts and Chévez-Barrios [12]	2015	Case report	Skin	Dual ANCA+
Bucur et al. [13]	2023	Systematic review	Renal, pulmonary	Dual ANCA+
Lötscher et al. [14]	2019	Case review	Renal	Dual ANCA+
Patnaik et al. [15]	2015	Case report	Skin	Dual ANCA+
Kunzler et al. [16]	2018	Case report	Skin	ANCA-
Mudoni et al. [17]	2018	Case report	Renal, muscle	Not reported
Jin et al. [18]	2018	Review	Renal, pulmonary	Dual ANCA+

Reviews such as Yaseen et al. [1] and Iorio et al. [2] provided overviews of systemic, renal, and pulmonary manifestations, reinforcing the high prevalence of dual ANCA positivity. Case reports, including Patnaik et al. [15] and Kunzler et al. [16], illustrated severe cutaneous necrosis and variable ANCA patterns. Case series, like Subesinghe et al. [11], documented both renal and pulmonary diseases with high relapse rates in those continuing cocaine use. The largest systematic review (Bucur et al. [13]) pooled over 100 patients and confirmed the skin and kidney as the most frequently involved organs. Treatment regimens were heterogeneous but most commonly initiated with corticosteroids, often escalated to rituximab or cyclophosphamide for severe or refractory disease. Plasmapheresis was used in fulminant cases, particularly with DAH or RPGN. Outcomes were favorable in most patients achieving drug cessation, while ongoing cocaine use was strongly linked to relapse. RoB analysis indicated that most evidence came from low-level observational reports, with frequent underreporting of drug cessation status and inconsistent ANCA testing. Nevertheless, recurrent findings across multiple studies strengthened the reliability of clinical patterns described. Eighteen studies were included after screening (see Figure 1 for PRISMA), comprising narrative reviews [1-5,7-10,18], a systematic review [13], two case series [11], and multiple single-patient case reports highlighting sentinel presentations [12,15-17]. Collectively, these sources describe a consistent phenotype of cocaine- and/or levamisole-associated vasculitis with prominent cutaneous, renal, and pulmonary involvement. Cutaneous disease-often retiform purpura or necrosis involving the ears and extremities-was repeatedly described across reviews and reports [2-4,12,15,16]. Renal involvement commonly manifested as RPGN, typically crescentic on biopsy when performed [11,13,14,18]. Pulmonary disease included DAH or alveolar infiltrates on imaging in severe cases [2,9,11,18]. Several sources also noted hematologic toxicity associated with levamisole exposure (e.g., neutropenia), which may coexist with vasculitic features [2-5].

Dual ANCA profile and diagnostics

A defining feature across the literature is dual ANCA positivity with both MPO-ANCA and PR3-ANCA detected in a substantial subset of cases, a pattern that should raise suspicion for levamisole adulteration when present alongside relevant exposure history [1-3,5,10,13,14,18]. Diagnostic confirmation varied: while some reports included comprehensive serologies, kidney or skin biopsy, and toxicology testing, others relied on clinical assessment and self-reported drug use, underscoring variability in case ascertainment [1-3,11-14,18]. Table 2 summarizes commonly used diagnostic modalities (ANCA testing, biopsy, and targeted imaging).

**Table 2:** Diagnostic tools. ANCA: antineutrophil cytoplasmic antibody

Author(s)	Diagnostic tools
Yaseen et al. [1]	ANCA, biopsy
Iorio et al. [2]	ANCA, imaging
Marquez et al. [3]	ANCA, biopsy
Berman et al. [4]	Biopsy
Ruffer et al. [5]	ANCA, imaging
Misra et al. [6]	ANCA
Younger [7]	Imaging
Puac-Polanco et al. [8]	Imaging
Underner et al. [9]	Imaging
Grau [10]	ANCA
Subesinghe et al. [11]	ANCA, biopsy
Roberts and Chévez-Barrios [12]	Biopsy
Bucur et al. [13]	ANCA, imaging
Lötscher et al. [14]	ANCA, biopsy
Patnaik et al. [15]	ANCA
Kunzler et al. [16]	Biopsy
Mudoni et al. [17]	Labs, imaging
Jin et al. [18]	ANCA, biopsy

Yaseen et al. [1] and Iorio et al. [2] synthesize the broader landscape of DIV and delineate the CLIV spectrum, emphasizing dual ANCA patterns and multiorgan involvement. Marquez et al. [3] and Berman et al. [4] detail cutaneous necrosis and classic auricular involvement. Ruffer et al. [5] discuss systemic features and reinforce the importance of exposure history. Misra et al. [6] focus on immunopathologic mechanisms relevant to AAV. Younger [7] and Puac-Polanco et al. [8] address neurologic/CNS and imaging considerations, respectively, while Underner et al. [9] highlight pulmonary manifestations. Grau [10] provides an earlier review framing dual ANCA as a diagnostic clue. Subesinghe et al. [11] present a case series with renal and pulmonary disease and note relapse risk with continued cocaine use. Roberts and Chévez-Barrios [12] describe a prototypical cutaneous case. Bucur et al. [13] offer a systematic review synthesizing organ involvement patterns, and Lötscher et al. [14] concentrate on renal disease. Patnaik et al. [15] and Kunzler et al. [16] report severe skin presentations (including ANCA-negative variants), Mudoni et al. [17] expand the spectrum to myopathic involvement, and Jin et al. [18] review renal-pulmonary overlap. Initial management frequently involved high-dose corticosteroids, with escalation to rituximab or cyclophosphamide in organ-threatening disease; plasmapheresis was used in fulminant RPGN/DAH [2,11,13,14,18]. Across reports, sustained abstinence from cocaine/levamisole was repeatedly linked to improved outcomes and lower relapse risk, whereas ongoing use correlated with recurrence [2,3,11,13]. Table 3 summarizes reported regimens and outcomes with source-level citations that favor immunosuppressive therapy in appropriately selected patients.

**Table 3:** Treatment and outcomes.

Author(s)	Treatment	Outcome
Yaseen et al. [1]	Immunosuppressants	Improved
Iorio et al. [2]	Steroids, rituximab	Improved
Marquez et al. [3]	Steroids	Improved
Berman et al. [4]	Steroids	Improved
Ruffer et al. [5]	Steroids, rituximab	Improved
Misra et al. [6]	Steroids	Improved
Younger [7]	Steroids	Improved
Puac-Polanco et al. [8]	None specified	Not specified
Underner et al. [9]	Steroids	Improved
Grau [10]	Steroids	Improved
Subesinghe et al. [11]	Steroids, plasmapheresis	Improved
Roberts and Chévez-Barrios [12]	Steroids	Improved
Bucur et al. [13]	Steroids, plasmapheresis	Improved
Lötscher et al. [14]	Steroids	Improved
Patnaik et al. [15]	Steroids	Improved
Kunzler et al. [16]	Steroids	Improved
Mudoni et al. [17]	IV fluids, supportive	Recovered
Jin et al. [18]	Steroids, rituximab	Improved

Given the heterogeneous designs and limited comparative data, we did not perform quantitative pooling and instead present a structured narrative synthesis. Consistent with Table 2, most studies incorporated ANCA serology (MPO and PR3), with biopsy (skin or kidney) when feasible to confirm small-vessel vasculitis or crescentic glomerulonephritis (GN); imaging (e.g., chest radiography/CT) supported DAH or pulmonary involvement when suspected [1-3,9-12,14,18]. Urine toxicology and explicit documentation of exposure were variably reported, affecting case certainty in several reports [2,3,11,13].

RoB analysis

The majority of included studies were case reports and small case series, which inherently carry a high risk of selection and publication bias, as they often highlight unusual or severe presentations while underreporting milder or self-limiting cases. Several reviews included in this analysis lacked explicit search strategies or inclusion criteria, increasing the likelihood of selection bias. Across studies, there was substantial heterogeneity in diagnostic confirmation-while some patients underwent comprehensive ANCA testing (both MPO and PR3), others were diagnosed solely on clinical grounds without confirmatory laboratory data, introducing potential misclassification bias. Reporting bias was common, with incomplete documentation of treatment regimens, follow-up duration, and patient outcomes. Moreover, drug cessation status-a key determinant of prognosis-was inconsistently reported, limiting interpretation of long-term outcomes. No included study was a randomized controlled trial; thus, the certainty of evidence regarding treatment efficacy remains low to very low according to the GRADE criteria. Despite these limitations, the recurrence of consistent clinical patterns-particularly dual ANCA positivity with concurrent renal and pulmonary involvement-across diverse study designs and settings supports the validity of the observed associations.

Discussion

CLIV remains underrecognized, despite its rising prevalence due to widespread cocaine adulteration. This systematic review reinforces the clinical importance of recognizing CLIV in patients presenting with dual ANCA positivity [2,3,5], especially when accompanied by RPGN and DAH [13,14]. The pathophysiology of CLIV is multifactorial. Levamisole, an immunomodulatory agent [2,3,18], induces vasculitis through neutrophil activation, NETosis, and autoantibody production [6], often resulting in dual ANCA (MPO and PR3) positivity. Cocaine itself can contribute to endothelial injury and promote autoimmunity. The overlap of these mechanisms explains the aggressive nature of CLIV and its rapid progression to renal and pulmonary involvement. Clinically, CLIV mimics idiopathic AAV but differs in patient demographics and exposure history. Young to middle-aged adults with a history of substance use are commonly affected. Cutaneous manifestations such as retiform purpura [12,15,16] and skin necrosis, especially involving the ears and extremities, are frequently observed. Renal biopsy often reveals crescentic GN [13,14], while lung imaging may show diffuse alveolar infiltrates [9] due to hemorrhage. Diagnostic workup should include detailed substance use history, ANCA serologies, urine drug screen [2,3], and confirmatory biopsy where feasible. The presence of both MPO-ANCA and PR3-ANCA is highly suggestive of levamisole involvement. Management hinges on cessation of cocaine/levamisole exposure and immunosuppression. Steroids remain first-line, while severe cases may require rituximab, cyclophosphamide, and plasmapheresis [13,14]. Supportive care, including dialysis or mechanical ventilation, is essential in organ-threatening disease. Relapse is common in patients who continue cocaine use. Long-term outcomes depend on sustained abstinence, emphasizing the need for addiction counseling [2,3].

## Conclusions

CLIV is a serious but potentially reversible vasculitis syndrome that requires prompt recognition and intervention. Early identification in cocaine users with suggestive clinical features-particularly dual ANCA positivity, RPGN, and DAH-is critical to preventing irreversible organ damage. Successful management hinges on both targeted immunosuppression and sustained cessation of cocaine/levamisole use. Multidisciplinary care involving nephrology, rheumatology, pulmonology, and addiction services is recommended to optimize patient outcomes. Long-term follow-up is essential to monitor for relapses and address the psychosocial components of substance use, which remain integral to preventing recurrence.

## Appendices

Supplemental material 1 

**Table 4:** Search log.

Database	Date_Run	Search_String	Hits
PubMed	2024-07-31	((levamisole[Title/Abstract] OR levamisole-adulterated[Title/Abstract]) AND cocaine[Title/Abstract]) AND (vasculitis[Title/Abstract] OR vasculopathy[Title/Abstract] OR ANCA[Title/Abstract]) Filters: Humans, English, 2015–2024	182
Embase	2024-07-31	("levamisole"/exp OR "levamisole-adulterated") AND "cocaine"/exp AND ("vasculitis"/exp OR "vasculopathy"/exp OR "ANCA"/exp) Filters: Humans, English, 2015–2024	94
Scopus	2024-07-31	TITLE-ABS-KEY(levamisole OR "levamisole-adulterated") AND TITLE-ABS-KEY(cocaine) AND TITLE-ABS-KEY(vasculitis OR vasculopathy OR ANCA) AND PUBYEAR > 2014	36
CENTRAL	2024-07-31	"levamisole AND cocaine AND vasculitis" (CENTRAL, 2015–2024)	0

Supplemental material 2 

**Table 5:** Citation overlap matrix.

Primary study	Yaseen et al. [1]	Iorio et al. [2]	Ruffer et al. [5]	Bucur et al. [13]
Marquez et al. [3]	✓	✓	✓	✓
Subesinghe et al. [11]	✓	✓	✓	✓
Roberts and Chévez-Barrios [12]	✓	✓	✓	✓
Patnaik et al. [15]	✓	✓	✓	✓
Kunzler et al. [16]	✓	✓	✓	✓
Mudoni et al. [17]	✓	✓	✓	✓
Lötscher et al. [14]	✓	✓	✓	✓
Jin et al. [18]	✓	✓	✓	✓

Supplemental material 3 

**Table 6:** Risk of bias (RoB) & GRADE summary.

Risk of Bias: Case Reports/Series (JBI Domains)
Most case reports and series satisfied the criteria for clear patient demographics, clinical history, and diagnostic confirmation. Common limitations: lack of toxicology confirmation, incomplete follow-up, and underreporting of relapse/abstinence.
Risk of Bias: Non-comparative Data
ROBINS-I was planned for observational cohorts; no eligible comparative cohorts were identified. Therefore, risk of bias is considered high for confounding and selection.
GRADE Certainty Assessment
Certainty downgraded for risk of bias (case-report level evidence), inconsistency (heterogeneity in outcomes), and imprecision (small sample sizes). Overall certainty: low to very low for all outcomes.
